# Cannabinoids and Chronic Liver Diseases

**DOI:** 10.3390/ijms23169423

**Published:** 2022-08-20

**Authors:** Ralph-Sydney Mboumba Bouassa, Giada Sebastiani, Vincenzo Di Marzo, Mohammad-Ali Jenabian, Cecilia T. Costiniuk

**Affiliations:** 1 Department of Biological Sciences and CERMO-FC Research Centre, Université du Québec à Montréal, Montreal, QC H2X 1Y4, Canada; 2 Infectious Diseases and Immunity in Global Health Program, Research Institute of McGill University Health Centre, Montreal, QC H3A 3J1, Canada; 3 Department of Medicine, Division of Gastroenterology and Hepatology, McGill University Health Centre, Montreal, QC H3A 3J1, Canada; 4 Department of Medicine, Division of Infectious Diseases and Chronic Viral Illness Service, Montreal, QC H3A 3J1, Canada; 5 Centre de Recherche de l’Institut Universitaire de Cardiologie et de Pneumologie de Québec, Département of Médecine, Faculté de Médecine, Université Laval, Quebec City, QC G1V 0A6, Canada; 6 Canada Excellence Research Chair on the Microbiome-Endocannabinoidome Axis in Metabolic Health (CERC-MEND), Université Laval, Quebec City, QC G1V 0A6, Canada; 7 Endocannabinoid Research Group, Institute of Biomolecular Chemistry, Consiglio Nazionale Delle Ricerche (CNR), 80078 Pozzuoli, Italy; 8 Institut sur la Nutrition et les Aliments Fonctionnels, Centre NUTRISS, École de Nutrition, Faculté des Sciences de L’agriculture et de L’alimentation, Université Laval, Quebec City, QC G1V 0A6, Canada; 9 Department of Microbiology and Immunology, McGill University, Montreal, QC H3A 3J1, Canada

**Keywords:** endocannabinoid system, cannabinoids, cannabidiol (CBD), tetrahydrocannabinol (THC), insulin resistance, chronic liver diseases, steatosis, NAFLD, NASH, ALD, HCV, HIV

## Abstract

Nonalcoholic fatty liver disease (NAFLD), alcohol-induced liver disease (ALD), and viral hepatitis are the main causes of morbidity and mortality related to chronic liver diseases (CLDs) worldwide. New therapeutic approaches to prevent or reverse these liver disorders are thus emerging. Although their etiologies differ, these CLDs all have in common a significant dysregulation of liver metabolism that is closely linked to the perturbation of the hepatic endocannabinoid system (eCBS) and inflammatory pathways. Therefore, targeting the hepatic eCBS might have promising therapeutic potential to overcome CLDs. Experimental models of CLDs and observational studies in humans suggest that cannabis and its derivatives may exert hepatoprotective effects against CLDs through diverse pathways. However, these promising therapeutic benefits are not yet fully validated, as the few completed clinical trials on phytocannabinoids, which are thought to hold the most promising therapeutic potential (cannabidiol or tetrahydrocannabivarin), remained inconclusive. Therefore, expanding research on less studied phytocannabinoids and their derivatives, with a focus on their mode of action on liver metabolism, might provide promising advances in the development of new and original therapeutics for the management of CLDs, such as NAFLD, ALD, or even hepatitis C-induced liver disorders.

## 1. Introduction

Accounting for nearly 2 million deaths per year worldwide, chronic liver diseases (CLDs) constitute a leading cause of mortality [1]. CLDs encompass a wide range of diseases with varied etiologies, including, but not limited to, nonalcoholic fatty liver diseases (NAFLDs), alcohol-related liver disease (ALD), and chronic infection with the hepatitis B (HBV) and C (HCV) viruses, which are all conditions that may progress to fibrosis, cirrhosis, and hepatocellular carcinoma (HCC) if untreated [1,2]. NAFLD is the most common CLD, with an estimated prevalence of from 25 to 30% worldwide, and it is driven by increasing rates of diet-induced obesity and diabetes [1,2,3]. NAFLD is characterized by the development of insulin resistance not linked to alcohol intake or other steatogenic medication [1,2,3], resulting in increased adipocyte lipolysis and high circulating free fatty acids and leading to steatosis, which is a pathologic accumulation of fat in the liver [3]. Insulin resistance occurring in NAFLD also leads to reduced hepatic glycogen storage and increased gluconeogenesis [3]. Nonalcoholic steatohepatitis (NASH) is a progressive clinical and pathological entity that is encompassed in the NAFLD spectrum and is characterized by liver histology, revealing also the presence of liver inflammation and swelling with hepatocyte injury. NASH can thus progress to liver fibrosis, cirrhosis, and HCC [1]. Meanwhile, prolonged alcohol abuse causes the development of ALD through complex signaling pathways by perturbating the immune response at the hepatic site with an aberrant activation of Kupffer cells, which secrete high amounts of proinflammatory cytokines, such as tumor necrosis factor alpha (TNF-α), and the recruitment of other innate immune cells at the hepatic site [4]. Alcoholic liver intoxication is also accompanied by mitochondrial dysfunctions and an increase in oxidative stress with the production of reactive oxygen species (ROS), which all lead to the establishment of a deleterious inflammatory microenvironment in the liver and the apoptosis of hepatocytes [4]. This liver inflammation is also accompanied by the progressive dysfunction of liver metabolism with an increase in abnormal lipogenesis, which promotes steatosis and acute hepatitis cirrhosis [2]. ALD gives rise to most liver-related deaths, with excess alcohol consumption accounting for over 50% of the cirrhosis-associated mortality worldwide [2].

Besides the alarming burden of NAFLD and ALD, around 350 million people were living with HBV (296 million) and HCV (58 million) in 2019, resulting in about 820,000 (HBV) and 290,000 (HCV) deaths, and mostly due to cirrhosis and HCC [5]. Indeed, similar to other CLDs, chronic viral hepatitis is also characterized by a persistent hepatic necroinflammation accompanied by hepatic metabolic dysfunctions, which favor the development of cirrhosis and cancer [6]. Despite the extensive effort to understand the pathophysiology of NAFLD and ALD, CLDs remain a persistent global health concern, and the functional therapeutic options are very limited. Apart from drastic lifestyle changes, with a reduction in fat and sugar intake, alcohol abstinence, and weight loss, the only pharmacological therapies approved to date remain vitamin E and pioglitazone, which are mostly reserved for patients with NASH and advanced liver fibrosis [7,8].

Due to their shared pathophysiology involving disturbing the liver metabolism and deleterious liver inflammation, coupled with the growing body of literature that suggests that the endocannabinoid system (eCBS) plays a key role in CLD pathogenesis, this signaling system may therefore constitute a potential therapeutic target in the management of these chronic inflammatory and metabolic diseases [9]. Herein, we discuss the latest evidence supporting the implication of the eCBS in the pathophysiology of CLDs, as well as the potential therapeutic impact of cannabis intake and cannabinoid-based medicines on the prognoses of CLDs.

## 2. Cannabinoids and the Endocannabinoid System (eCBS)

### 2.1. The eCBS

The eCBS is a complex cell-signaling system that is widespread in almost all mammalian organs and tissues, where it regulates several physiological functions (see reference [10] for full detailed review). Briefly, the eCBS is composed of cannabinoid receptors (CBRs), among which the most studied are cannabinoid receptors 1 (CB1R) and 2 (CB2R), their endogenous lipid ligands, the so-called “endocannabinoids (eCB)”: anandamide (AEA) and 2-arachidonoyl glycerol (2-AG), as well as their most studied synthesizing enzymes: *N*-acyltransferase and *N*-acyl-phosphatidylethanolamines-specific phospholipase D for AEA, and phospholipase Cβ and diacylglycerol (DAG) lipases α and β for 2-AG. Finally, their main catabolic enzymes are fatty acid amide hydrolase (FAAH) and monoacylglycerol lipase for AEA and 2-AG, respectively [10,11,12]. It has also been reported that eCB can be oxidized by the cyclooxygenase-2, lipoxygenases, and even the cytochromes P450 [10,11,12]. These enzymes and pathways are often used to produce or degrade some eCB congeners, including, but not limited to, the AEA congeners, the *N*-acylethanolamines (NAEs), and the 2-AG congeners, the 2-monoacylglycerols (2-MAGs), which, however, in most cases, do not efficaciously activate CBRs, although they do modulate the activity of other eCB targets [13].

CBRs regulate important intracellular signal transduction pathways, comprising the activation of the phosphorylation of mitogen-activated protein kinases and the suppression of adenylyl cyclase activity and calcium channels [11]. In addition to CB1R and CB2R, the effects of endocannabinoids can also be mediated via other nonclassical cannabinoid receptors, including members of the orphan G protein-coupled receptors GPR18, GPR55, and GPR119, at least one member of the transient receptor potential vanilloid (TRPV) channel family, TRPV subtype 1 (TRPV-1), and some isoforms of the nuclear receptor family of peroxisome proliferator-activated receptors (PPARα and PPARγ) [10,11,12,14,15]. CB1R is highly abundant in the central nervous system (CNS), where it modulates physiological functions associated with the nervous system; in contrast, CB2R is mainly found on immune cells, and the stimulation of peripheral CB2R results in anti-inflammatory and immunomodulating effects, and thus plays a role in alleviating both inflammatory and neuropathic pains [11]. Both CB1R and CB2R have also been found in other organs and tissues, where they regulate, among other things, hormone secretion, blood circulation, gastrointestinal-tract functions, reproductive-system functions, and energy metabolism [16,17]. There is a growing body of literature supporting the implication of the eCBS in the regulation of hepatic lipid and glucose metabolisms [18,19] and the modulation of the immunological response at the hepatic site, and particularly in the establishment and regulation of liver inflammation [20,21]. Additionally, it has been shown that hepatic CB1R and CB2R have dual and opposite effects on fibrogenesis associated with chronic liver injury: CB1R promotes profibrogenic effects, while CB2R elicits antifibrogenic effects [22,23]. These observations suggest a differential distribution of these two cannabinoid receptors in the liver, and more particularly, between the different hepatic-cell types [24].

### 2.2. Phytocannabinoids

Besides eCB, cannabinoids isolated from the cannabis plant, referred to as phytocannabinoids, can also bind and modulate the activity of the eCBS [10,25]. While delta-9-tetrahydrocannabinol (THC) is the most abundant and the major psychoactive phytocannabinoid, cannabidiol (CBD) is the second most abundant and is the main non-psychoactive compound of the cannabis plant [10,25]. THC is a high-affinity partial agonist of both CB1R and CB2R [25]. Given that CB1R is highly expressed in the CNS, most of the popular effects exerted by THC include its psychotropic effects and impact on appetite stimulation [11,25]. THC also has anti-inflammatory properties by inhibiting the proinflammatory response and shifting the immune response toward an anti-inflammatory phenotype, inducing apoptosis, and suppressing cell proliferation, mainly through the activation of CB2R [26]. In addition to classical cannabinoid receptors, THC can also activate GPR55 and GPR18, isoforms of PPAR, as well as members of TRPV receptors [17,26].

Unlike THC, CBD is a non-psychoactive phytocannabinoid, and it possesses analgesic, neuroprotective, anticonvulsant, antiemetic, spasmolytic, and anti-inflammatory properties [11,26]. CBD has been proposed by some to act as a negative allosteric modulator of CB1R, and it has been shown to exert its anti-inflammatory properties through non-CBRs by inhibiting the activity of the NF-kappa B pathway, the activator protein-1 and nuclear factor of activated T-cell transcriptional activity, and leading to the suppression of proinflammatory cytokines (IL-2, TNF-α, INF-γ) and immune-cell activation and proliferation [26,27,28]. Moreover, CBD can induce the secretion of anti-inflammatory cytokines via the upregulation of STAT3 transcription factor, which governs the production of tumor growth factor beta (TGF-β), one of the main immunosuppressive cytokines [26]. As for THC, CBD has also been found to modulate the activity of nonclassical cannabinoid receptors, including GPRs and TRP channels (namely TRPV1, TRPV2, and TRPA1, which are activated, and TRPM8, which is inhibited), as well as PPARs [25]. Both THC and CBD have also been found to exert antioxidant activity by scavenging free ROS and blocking ROS generation [26]. Finally, the cannabis plant contains other less abundant phytocannabinoids, including tetrahydrocannabivarin (THCV), delta-9-tetrahydrocannabinolic acid (THCA), cannabidiolic acid (CBDA), and tetrahydrocannabivarinic acid (THCVA), which have been found to exert diverse biological activities, similar to CBD (Table 1) [25].

## 3. The Endocannabinoid System in Chronic Liver Diseases

### 3.1. Hepatic Endocannabinoid System in Normal Physiology

Evidence for the involvement of the eCBS in the normal physiology and functioning of the liver is scarce, and when it exists, it comes mainly from in vitro studies or animal models using the agonists and antagonists of cannabinoid receptors. CB1R is considered a primary cannabinoid receptor in the liver and is located on hepatic sinusoidal cells, stellate cells, and hepatocytes, whereas CB2R is mostly localized in Kupffer and hepatic stellate cells [43]. The eCBS has emerged as a regulator of several aspects of liver pathophysiology [44]. Under normal physiological conditions, the expressions of the major mediators of the eCBS signaling pathways, CB1R and CB2R, and their endogenous ligands (AEA and 2-AG), are detected only in low levels in liver tissues [17]. This would, at least in part, be the result of the high hepatocellular expression of the FAAH, which is one of the most important enzymes for endocannabinoid ligand catabolism [45]. These observations suggest that hepatic metabolic pathways regulated by the eCBS in normal physiology required, at most, only a low activation level of this system. One exception to this rule is represented by the regulation of the cell-cycle progression via CB1R in the regenerating liver, when AEA is overproduced [16,46].

Indeed, using zebrafish and the genetic or chemical inhibition of cannabinoid receptors, Liu et al. demonstrated the involvement of the eCBS very early in the regulation of liver development and metabolic functions [47]. Indeed, they showed that exposing zebrafish embryos to specific CB1R agonists (O2545 or leelamine hydrochloride) and a CB2R agonist (JWH015) early during the first hours of their development resulted in an increased size of the embryo livers, whereas CB1R-antagonist (rimonabant) or CB2R-antagonist (AM630) exposure reduced the liver size. In addition, when *cnr1* and *cnr2* genes were altered but not completely silenced in zebrafish embryos, the liver development was also negatively affected, resulting in smaller livers with reduced number of hepatocytes and their differentiation. Together, these data suggest that CB1R and CB2R are required for normal hepatogenesis and, by extension, highlight the crucial role of the eCBS in normal liver development [47]. In a model of genetically induced obese mice, *cnr1* knockout mice showed severe growth retardation, and they were more glucose intolerant than obese mice. Antagonizing CB1R in obese mice had no effect on the glucose metabolism, suggesting a possible requirement for CB1R signaling to promote growth and regulate normal glucose homeostasis in development [48].

Apart from liver development and lipid metabolism alterations, Liu et al. have also uncovered the significant involvement of the eCBS in the metabolism of amino acids, and especially methionine metabolism, which was significantly altered in CB2R and CB1R knockout zebrafish compared to the wild type [47]. Consistent with what was observed by Liu et al., using human-derived immortalized hepatocytes, De Gottardi et al. also showed that both CB1R and CB2R played important roles in hepatic lipid metabolism by regulating the expressions of the key hepatic enzymes of lipid synthesis and transport, such as carnitine palmitoyltransferase 1, sterol regulatory element binding protein, and its targets acetyl-CoA carboxylase-1 and fatty acid synthase, as well as lecithin–cholesterol acetyltransferase [49]. In another study, Chanda et al. elucidated the critical role of CB1R in the regulation of hepatic lipid metabolism by dysregulating hepatic insulin-receptor signaling via the endoplasmic-reticulum-bound transcription factor cyclic AMP-response element-binding protein H (CREBH)-dependent pathway. Indeed, treating mice liver with the CB1R agonist 2-AG, or the overexpression of CREBH in mouse hepatocytes with adenoviral transfection, resulted in the increased expression of the hepatic DAG level and the phosphorylation of protein kinase Cε, which, in turn, inhibited hepatic insulin-receptor signaling. In addition, knocking down CREBH, or using a CB1R antagonist (AM251), restored the insulin-receptor signaling, demonstrating the involvement of CB1R in the hepatic lipid metabolism through the modulation of hepatic insulin-receptor signaling via a CREBH-dependent pathway [50,51]. Similar to lipid metabolism, Chanda et al. also evidenced the critical role of CB1R in the regulation of hepatic glucose metabolism, always under the control of the CREBH pathway, in both rat and human primary hepatocytes. Indeed, CB1R activation by 2-AG specifically promoted CREBH gene expression via the phosphorylation of the JNK signaling pathway and c-Jun binding to the AP-1 binding site in the CREBH gene promoter, resulting in a significant increase in the expressions of the key genes coding for gluconeogenic enzymes phosphoenolpyruvate carboxykinase and glucose-6-phosphatase catalytic subunit, leading to glucose production in primary hepatocytes. Knocking down CREBH, or using a CB1R antagonist (AM251) or insulin treatment, significantly reduced the gluconeogenic gene expression and decreased the hepatic gluconeogenesis in both rat and human primary hepatocytes [50,51]. Altogether, these findings demonstrate that the hepatic eCBS, although expressed at a lower level in the liver compared with other organs, contributes to the normal development of the liver morphology, and orchestrates crucial metabolic functions in the liver.

### 3.2. Hepatic Endocannabinoid System in NAFLD and NASH

NAFLD is the most prevalent CLD (from 25 to 30% worldwide), and it constitutes a common comorbidity in individuals suffering from metabolic syndrome [1,2,3]. NAFLD is characterized by increased adipocyte lipolysis and high circulating free fatty acids, leading to a pathologic accumulation of fat in the liver (steatosis), caused primarily by insulin resistance and not linked to alcohol intake or other steatogenic medication [1,2,3]. Insulin resistance occurring in NAFLD also leads to reduced hepatic glycogen storage and increased gluconeogenesis [3]. NASH is defined by the presence of liver steatosis accompanied by hepatocyte apoptosis exacerbation and swelling, liver inflammation with the infiltration and activation of proinflammatory immune cells in the liver, and injury [3]. One of the key inflammatory pathways involved in NASH is pyroptosis [52]. Pyroptosis is an inflammatory programmed-cell-death pathway, triggered by various pathological stimuli, such as pathogen-associated molecular patterns, or damage-associated molecular patterns (DAMPs) generated from damaged tissues [52]. Pyroptosis can occur in hepatocytes as well as in Kupffer cells, and it is mediated by the activation of the NOD-like receptor pyrin domain containing 3 (NLRP3) inflammasome by DAMPs generated from damaged hepatocytes [52]. The activated NLRP3 inflammasome will then activate the caspase-1 cascade, resulting in, on the one hand, the maturation and release of proinflammatory cytokines (IL-1β and IL-18) in the hepatic environment, and, on the other hand, to gasdermin D-mediated pyroptotic cell death, thus exacerbating the liver injury and inflammation [52]. It is therefore necessary to elucidate the role played by the eCBS in the pathological processes related to NAFLD and NASH, such as hepatic steatosis, insulin resistance, hepatic apoptosis, or inflammatory pyroptosis, in order to identify potential cannabinoid-associated therapeutic targets.

Several lines of evidence suggest the crucial role of the hepatic eCBS in the pathogenesis of NAFLD [53,54]. Given that CB1R is one of the major components of the liver eCBS, its modulation is intimately involved in chronic liver-metabolism disorders [53,54]. Accordingly, Cota et al. showed, in a mouse model of high fat diet (HFD)-induced obesity, that the hepatic expression of CB1R was significantly increased in wild-type mice fed an HFD compared with those fed a normal diet, suggesting that the increase level of CB1R observed in NAFLD is due to fat-metabolism disorders [55]. Other studies also confirmed the implication of CB1R in the pathogenesis of NAFLD, as the activation of hepatic CB1R triggered lipogenesis pathways in the liver, as well as monounsaturated fatty acid accumulation and the inhibition of FAAH, which was followed by an increased level of AEA, resulting in the setting of steatosis and insulin resistance, which are the main causes of NAFLD [49,55,56,57,58]. Interestingly, the blocking of peripheral or hepatic CB1R inhibited hepatic steatosis and insulin resistance while improving several biomarkers associated with NAFLD and NASH [59,60,61]. Indeed, Khan et al. showed that, in addition to the improvement in NAFLD, HFD-fed mice treated with a selective antagonist of peripheral CB1R also had a strong reduction in the biomarkers of liver injury and inflammation associated with NASH, as demonstrated by a significant decrease in circulating transaminases alanine aminotransferase (ALT), aspartate aminotransferase (AST), and lactate dehydrogenase (LDH), compared with HFD-fed mice not treated with the CB1R antagonist [60]. Likewise, Osei-Hyiaman et al. showed that knocking out hepatic CB1R in mice was accompanied by reduced hepatic fatty accumulation, insulin resistance, and lipogenesis compared with wild-type mice [57,58].

**Figure 1:**
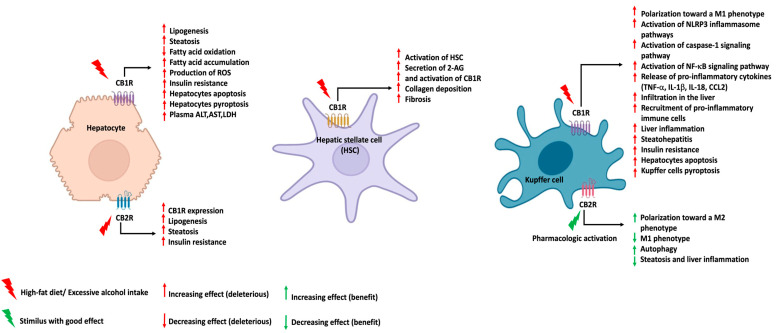
Effects of activation of hepatic cannabinoid receptors CB1R and CB2R on chronic liver diseases. The effects of CB1R and CB2R activation by different stimuli, such as high-fat diet, excessive alcohol intake, or pharmacological activation, on the main processes involved in chronic liver pathologies, including nonalcoholic fatty liver disease, nonalcoholic steatohepatitis, and alcohol-associated liver disease. These CB1R/CB2R-mediated biological effects are presented for hepatocytes, hepatic stellate cells, and Kupffer cells.

Pyroptosis, which, as mentioned above, is a form of cell death that results from the activation of the NLRP3 inflammasome pathway in Kupffer cells, represents a key process in the progression of NAFLD to NASH, as shown in Yu et al. [52] in a mouse model of liver inflammation. Yang et al. showed that the hepatic CB1R expression was significantly increased and strongly correlated with the expression of the NLRP3 inflammasome, and particularly in the Kupffer cells of mice treated with carbon tetrachloride (CCl_4_) and methionine-choline-deficient and high fat (MCDHF) diets [62]. In addition, while the activation of the CB1R with an agonist promoted the expression and activation of the NLRP3 inflammasome in Kupffer cells, blocking CB1R with the antagonist AM281 reduced the expression of NLRP3, inflammasome activation, and liver inflammation [62]. Accordingly, increased levels of hepatic CB1R expression constitute both a consequence, but also a cause, of NAFLD and NASH development, and therefore, specifically targeting both peripheral and hepatic CB1R could constitute a promising therapeutic strategy to target NAFLD (Figure 1). However, a recent study conducted in mice lacking hepatic CB1R and fed an HFD showed that CB1R deletion did not prevent the development of NAFLD, nor did it protect the mice from experimental liver fibrosis [63]. These latest results, while critically examined by Kunos et al. [64], may yet underscore the fact that NAFLD development is not exclusively restricted to CB1R signaling, and that other CB1R-independent pathways also participate in the pathogenesis of NAFLD.

In conjunction with the crucial role of CB1R in NAFLD, mounting evidence demonstrates the role of CB2R in the establishment of liver steatosis and insulin resistance. Indeed, treating HFD-fed mice with the CB2R agonist JWH-133 potentiated liver inflammation, insulin resistance, and hepatic steatosis, supporting the role of CB2R in the setting of NAFLD, as well as the progression to NASH [65]. Furthermore, knocking out CB2R in HFD-fed mice resulted in a decreased infiltration of macrophage in adipose tissues, along with a reduction in the expressions of *Tnf* and *Ccl2*, which are two genes that code for proinflammatory cytokines associated with the establishment of liver as well as adipose-tissue inflammation [65]. Aligned with these findings, Agudo et al. also showed that antagonizing CB2R in HFD-fed mice resulted in improved insulin sensitivity, and knocking out CB2R in HFD-fed mice resulted in improvements in steatosis and insulin resistance, despite increases in food intake and body weight with age [66]. Interestingly, in the human liver cell line HepG2, the activation of CB2R significantly modulated lipogenesis and induced the increased expression of CB1R, indicating that, beyond its direct contribution in the setting of NAFLD and NASH, the activation of hepatic CB2R would also potentiate the deleterious effects of hepatic CB1R, thus creating a vicious cycle [49]. Together, these findings suggest that CB2R could promote steatosis directly by inducing liver inflammation, contributing to NASH, and indirectly by increasing the hepatic CB1R expression, thereby contributing to NAFLD, NASH, and liver fibrosis. These findings may suggest that antagonizing CB2R may also represent a promising therapeutic approach for the management of NAFLD and NASH (Figure 1). However, these findings are also in sharp contrast with several other data in the literature that indicate that the eCB action at CB2R instead reduces liver inflammation and fibrosis, as well as cirrhosis, via several molecular mechanisms [50,67,68,69,70], some of which are similar to those proposed to mediate the protective actions of this receptors against ALD (see below) and in other inflammatory conditions. Therefore, any decision regarding the clinical use of CB2R agonists or antagonists in NAFLD and NASH should await further studies. Aside from the classical cannabinoid receptors, PPAR isoforms (PPAR-α, PPAR-β/δ, and PPAR-γ) have also been found to play key roles in in vitro and mice models of NAFLD by regulating adipogenesis and inhibiting liver fibrosis [40,50]. Moreover, currently, one of the only treatments for NASH and advanced liver fibrosis in humans is pioglitazone, a PPAR-γ agonist, which thus demonstrates the promising potential of treatments that target these receptors in managing NAFLD and NASH [8].

### 3.3. Hepatic Endocannabinoid System in ALD

More than half of the cirrhosis-associated mortality around the world is caused by prolonged alcohol abuse, making ALD the main cause of liver-related deaths worldwide [1]. Similar to NAFLD, ALD also comprises symptoms including liver injury accompanied by simple steatosis or steatohepatitis, which can progress to liver cirrhosis and HCC [24]. Indeed, as for HFD, excessive and chronic alcohol consumption causes hepatic immune disturbances, and the excessive activation of inflammatory caspases in proinflammatory Kupffer cells, resulting in pyroptosis with the secretion of inflammatory cytokines, and oxidative stress with the production of ROS, and it is accompanied by the metabolic dysfunction of hepatocytes and abnormal lipogenesis, which leads to hepatic steatosis [4,24]. Given the underlying similarities in ALD and NAFLD/NASH pathogenesis, it is not surprising that alcohol intake can also induce changes in the eCBS [71]. Indeed, it has been shown that the hepatic eCBS plays a crucial role in the onset and progression of alcohol-induced hepatic steatosis and steatohepatitis [72]. Patsenker et al. showed a high expression of hepatic CB1R in the fibrotic livers of patients with alcohol-associated liver cirrhosis, and hepatic CB1R inhibition suppressed the HSC activation, in turn reducing hepatic inflammation and alcoholic liver fibrosis [72]. In a model of alcohol-induced steatosis, Jeong et al. also reported significant increases in the levels of hepatic CB1R and 2-AG in mice exposed to alcohol [73]. In addition, HSCs isolated from mice exposed to alcohol were able to stimulate the increase in the expression of lipogenic enzymes in hepatocytes in vitro. Moreover, it has also been shown that, in mice exposed to alcohol, the deletion of CB1R, as well as the pharmacologic blockage of CB1R with a nonselective CB1R antagonist, such as SR141716 (the rimonabant drug), or a peripheral selective CB1R antagonist, was found to significantly reduce alcohol-induced liver steatosis [73,74]. Conversely, similar to what was observed in a mouse model of NASH [62], Jiang et al. showed that the feeding of mice with excessive alcohol promoted caspase-1-induced pyroptosis in Kupffer cells, with the activation of the NLRP3 inflammasome and the release of a high amount of proinflammatory cytokines, which thus exacerbated the liver inflammation [34]. Treating alcohol-fed mice with CBD inhibited the activation of NLRP3 inflammasome, reduced the pyroptosis and liver inflammation, and protected the mice livers against steatohepatitis [34]. Accordingly, these findings suggest that, by activating hepatic CB1R, chronic alcohol intake stimulates the secretion of 2-AG by activated HSCs, which, in turn, promotes hepatic lipogenesis, on the one hand, and, on the other hand, inhibits fatty acid oxidation, thereby creating a disbalance between lipogenesis and lipid oxidation and resulting in chronic fat accumulation in the liver and steatosis [4,71,72,73]. Moreover, the activation of the hepatic CB1R by excessive alcohol intake also drives the activation of the key processing pathways involved in the setting of alcohol-induced steatohepatitis and liver inflammation [34]. Just as for NAFLD, these preclinical findings emphasize the potential role of CB1R in the worsening process of ALD, making it a potential therapeutic target to alleviate ALD (Figure 1).

In contrast to the deleterious effects exerted by hepatic CB1R in the occurrence of ALD, several studies have demonstrated that the activation of hepatic CB2R exerts a protective role against ALD [72]. Indeed, alcohol can polarize Kupffer cells to a proinflammatory M1 phenotype, which secretes a large amount of inflammatory cytokines, such as tumor necrosis factor (TNF), the main mediator of alcohol-induced liver damage, through the increase in hepatic lipogenesis and the inhibition of fatty acid oxidation [4]. Accordingly, by treating alcohol-fed mice with a CB2R-specific agonist (JWH-133), Louvet et al. observed an improvement in the alcoholic liver injury resulting from the polarization of Kupffer cells into a protective M2 anti-inflammatory phenotype [43]. In addition, CB2R activation was further found to play its protective role by inhibiting hepatic inflammation and steatosis in Kupffer cells through an autophagy-dependent pathway [75]. The critical protective role of CB2R against ALD was further evidenced in a mouse model of ALD, in which stuffing CB2R-deficient mice with alcohol induced increases in hepatic lipogenesis, inflammation accompanied by HSC activation, and collagen deposition [76]. These findings indicate that, in contrast to hepatic CB1R, CB2R plays a protective role against ALD by driving Kupffer-cell polarization toward an anti-inflammatory phenotype. Overall, these findings pinpoint the dual nature of the eCBS in the pathophysiology of ALD, suggesting a bifaceted strategy toward both an inhibition of hepatic CB1R and the activation of hepatic CB2R to counteract alcohol-related liver steatosis and inflammation (Figure 1).

### 3.4. The Hepatic Endocannabinoid System in Chronic Viral Hepatitis

Chronic viral hepatitis, and especially chronic hepatitis B and C infections, also account for a large burden of liver-related deaths worldwide (820,000 and 290,000 in 2019, respectively), which is mostly due to aggravation to cirrhosis and HCC [5]. Chronic viral hepatitis is characterized by a persistent hepatic necroinflammation, which is accompanied by hepatic metabolic dysfunctions [6]. Just as for the aforementioned liver pathologies, chronic viral hepatitis, and particularly chronic HCV infection, also induces the activation of the eCBS in the liver [77,78,79,80]. So far, only a few studies exist concerning the effect of HBV on the eCBS, and they show that HBV infection is able to increase the expression of CB1R in the liver, but to a lesser extent than HCV infection [79]. Unlike HBV, several reports have shown that HCV infection induces several changes in the eCBS [77,78,79,80]. In chronic HCV-infected individuals, van der Poorten et al. showed that HCV was able to increase the expression of hepatic CB1R specifically and significantly, and this increase was associated with the progression of the disease [79]. These observations were validated with an in vitro model of HCV-transfected hepatocytes, showing that the CB1R expression was significantly upregulated in HCV-transfected hepatocytes, and that this increase was specifically attributable to the presence of HCV structural proteins [79]. In line with these observations, Patsenker et al. also evidenced a significant activation of the eCBS during chronic HCV infection, with increased levels of both AEA and 2-AG in the plasma of chronically HCV-infected individuals, but not in the liver, accompanied by a decrease in the proinflammatory cytokine levels [80]. They also showed that HSC cocultured with PBMC from HCV-infected individuals underwent phenotypic changes to harbor a pro-fibrogenic phenotype, and that 2-AG but not AEA treatment was able to enhance this effect [80]. Based on these observations, they finally suggested that HCV infection could activate the peripheral eCBS, thereby weakening the immune response towards HCV, and worsening the disease by inducing fibrosis via the activation of HSC [80].

Finally, some recent studies showed how a genetic dysfunctional variant of the CB2R (single-nucleotide polymorphism (SNP) rs35761398) is associated with HCV-induced necroinflammation, with or without a concurrent infection with HIV [81,82], suggesting that this eCB receptor may instead play a protective role in this context.

## 4. Impact of Cannabis Intake and Cannabinoid-Based Medicine on NAFLD, NASH, ALD, and HCV-Induced Liver Disorders: Evidence from Preclinical, Observational, and Clinical-Trial Studies

### 4.1. Cannabis Use and CLD

#### 4.1.1. Cannabis Use and NAFLD and NASH

In a large population-based case–control study that aimed to assess the potential relationship between cannabis use and NAFLD, and including more than 5 millions patients in the United States, Adejumo et al. reported a significantly reduced prevalence of NAFLD in cannabis users (Table 2) [83].

Aligned with these findings, in another study, the authors found an inverse association between cannabis use and NAFLD in adult individuals [89]. In addition, Vázquez-Bourgon et al. also reported that cannabis users presented significantly lower fatty liver index scores, accompanied by less frequent liver steatosis, and a lower weight, body-mass index, total cholesterol, and low-density lipoprotein cholesterol compared with noncannabis users [97,100]. These beneficial effects of cannabis use were also confirmed in several other observational studies, highlighting a link between prolonged cannabis intake and an improvement in the strong predictors of NAFLD and NASH development, including insulin resistance [84,97], obesity, metabolic syndrome [85,86,87], and diabetes mellitus [88]. Furthermore, in a recent large two-sample mendelian randomization study, no significant causal effect between either lifetime cannabis use, cannabis-use dependence, or even cannabis-use disorder and the risk for NAFLD development were evidenced [101].

#### 4.1.2. Cannabis Use and ALD

Similar to what was observed for NAFLD, Adejumo et al. also found that, among alcohol users, those who additionally used cannabis had a significantly a lower risk of ALD, as evidenced by the lower prevalence of alcohol-induced steatosis, steatohepatitis, fibrosis, cirrhosis, and hepatocellular carcinoma [90]. In a recent case–control study, the authors found that alcohol users with alcohol-induced liver cirrhosis had a higher prevalence of diabetes and premorbid body-mass indexes compared with those without alcohol-induced cirrhosis, and the latest were more likely to use other substances, including cannabis [102]. Finally, in an exploratory study, the authors were not able to detect any predictive link between cannabis smoking and alcohol-induced liver fibrosis [91].

#### 4.1.3. Cannabis Use and HCV/HBV-Associated CLD

The impact of cannabis use has been largely evaluated in HCV-infected individuals (Table 2). Unlike NAFLD or ALD, studies on HCV-infected individuals seem to be conflicting (Table 2). Indeed, early observational studies argue in favor of the deleterious effects of cannabis use on the liver, with increased steatosis and severe fibrosis in chronically HCV-infected individuals [72]. In a cross-sectional study on 270 untreated chronically HCV-monoinfected individuals, Hézode et al. showed that daily cannabis smoking was an independent predictor of liver fibrosis progression and severe hepatic fibrosis [98]. Similarly, in 315 untreated chronically HCV-monoinfected individuals, Hézode et al. also showed that marked steatosis was more frequent in daily cannabis users compared with occasional users and nonusers [99]. Another study showed that daily compared with nondaily cannabis use was significantly associated with moderate to severe fibrosis in a cohort of 204 untreated chronically HCV-infected participants [103]. In contrast, more recent studies have consistently documented a significant reduced risk of insulin resistance, liver steatosis, inflammation, fibrosis, and cirrhosis in HCV-infected individuals who smoked cannabis compared to noncannabis users [90,92,93,94,95,96,97,104,105]. In a longitudinal evaluation on 703 HCV–HIV-coinfected individuals, Carrieri et al. showed that cannabis use was associated with a lower insulin-resistance risk [97]. Similar findings were observed in a five-year longitudinal study, with regular or daily cannabis use that was associated with an over 50% lower risk of elevated steatosis (fatty liver index) in 997 HCV–HIV-coinfected individuals [92], and with a reduced risk of diabetes [94]. Nordmann et al. also showed similar findings in a cross-sectional study of 838 HCV–HIV-coinfected participants, with a reduced prevalence of steatosis independently associated with daily cannabis use [95]. In addition, in a large cohort of 3706 HBV-infected individuals, Barré et al. showed that current cannabis use was associated with an over 50% lower risk of obesity compared with no lifetime use [93]. One of the main differences between these two groups of studies that may explain these conflicting findings is that the first studies were conducted in HCV-infected individuals receiving no treatment for HCV infection, whereas most of the others included mainly HCV-infected individuals receiving antiviral treatment. Indeed, some reports have shown that cannabis use, or cannabinoid-containing medicines, improve the retention and virological outcomes in patients treated for HCV [106,107]. In addition, most of the participants from the more recent studies were coinfected with HIV, and this factor could influence the physiological outcomes related to the eCBS [108]. On the other hand, earlier studies seemed to be biased by reverse causality, as suggested by Brunet et al. Indeed, Brunet et al. have suggested that changes in patient behavior caused by HCV-related advanced liver disease, such as an increase in cannabis intake to combat the symptoms of advanced liver disease, could induce a reverse-causation bias, falsely indicating that cannabis intake would have worsened the HCV-related liver disease. Therefore, to ensure the temporal causality association between cannabis smoking and the occurrence of worsened HCV-related liver disease, Brunet et al. evaluated 690 HIV–HCV-coinfected individuals without significant fibrosis or end-stage liver disease at baseline, and who reported cannabis use from 6 to 12 months before the assessment of the liver-disease progression. They found no evidence of a deleterious effect of cannabis intake on the progression of HCV infection to significant liver fibrosis or cirrhosis. Although they observed a slight increase in the risk of progression to cirrhosis with the increase in the cannabis intake, this risk was significantly lowered after lagging the cannabis exposure to from 6 to 12 months before the diagnosis of the liver-disease progression, thereby suggesting that the findings from the previous studies would have been affected by a reverse causation due to self-medication [104].

Overall, these studies demonstrate that the therapeutic use of cannabis for the management of CLDs, such as NAFLD, NASH, ALD, or end-stage liver diseases associated with chronic HCV infection, may be a promising strategy [95,97]. By interacting with the peripheral, or more specifically, the hepatic eCBS, the phytocannabinoids contained in cannabis preparations could counteract the deleterious effects, such as liver inflammation and energetic metabolism disorder, exerted by the various etiologies of CLDs [9]. However, several parameters associated with the possible medical use of cannabis for the management of liver diseases must be taken into account [109]. Indeed, the beneficial results observed in these studies were from people using cannabis of often unknown origin and potentially great differences in cannabinoid compositions, in a nonstandardized way, which makes it impossible to determine the optimal amount for therapeutic use in this context. In addition, these people smoked cannabis, which can clearly impact the bioavailability of active substances in the body, and beyond that, could induce deleterious effects on other organs, such as the lungs. Indeed, there is no mention of the strain of cannabis used or the level of active substance in the cannabis used in most of these studies. Given the large variability of cannabis strains, and their differences in cannabinoid-level contents, an eventual therapeutic use would also require standardization [109], and particularly in view of the fact that some noncannabinoid components, such as β-caryophyllene, may also exert protective effects by acting, in part, via CB2Rs [68,69]. Finally, another parameter to take into account is the genetic predisposition, as, depending on the populations and frequency of consumption, cannabis could have different effects [110], and particularly in view of the existence of SNPs in encoding genes that lead to dysfunctional CBRs (see above).

### 4.2. Cannabinoid-Based Medicine and CLDs

In this context, several studies have focused on the evaluation of isolated phytocannabinoids as therapeutic options against CLDs [111]. Although THC is the most abundant cannabinoid in cannabis, it is very plausible that the effects observed in CLDs are not attributable to this molecule, but rather to another compound or group of compounds. Indeed, THC is responsible for the increased appetite observed in cannabis users through the activation of CB1R, which induces increased food intake and, hence, may promote weight gain and, eventually, steatosis [112,113]. Moreover, THC treatment induces the significant accumulation of hepatic AEA and 2-AG by counteracting their enzymatic degradation and hydrolysis [114]. Given that these endocannabinoids are also upregulated in cirrhotic livers [115], one can therefore assume that the hepatoprotective effect of cannabis would not be mediated by THC, unless one assumes that the chronic administration of this molecule leads to the desensitization of CB1R, and subsequent protective effects on hepatocytes and against inflammation/fibrosis. Thus, because of its psychotropic effect and its stimulating effect on food intake and lipogenesis via CB1Rs, THC is less studied as a therapeutic option for managing CLDs.

Unlike THC, a growing body of evidence from preclinical studies has demonstrated that CBD represents the cannabinoid candidate with the most promising therapeutic potential for CLDs [72]. First, CBD acts as a noncompetitive negative allosteric modulator of CB1R, exerting weak agonist effects on this receptor, and therefore, it does not cause either the psychotropic effects of THC [116,117], or the depressive effects observed with rimonabant, which is an inverse antagonist of CB1R that was initially approved by the European Union for the treatment of obesity and its comorbidities, such as NAFLD and NASH [118]. Rimonabant demonstrated promising potential in clinical trials in humans as a treatment against obesity by decreasing fatty liver, fat accumulation in adipose tissue, and hepatic glucose production, and by increasing the hepatic insulin sensitivity [118]. However, the therapeutic effects of this drug were overshadowed by its severe psychiatric side effects, thus leading to its withdrawal from the market [118]. Moreover, CBD is also an inverse agonist for CB2R [117] and the nonclassical cannabinoid receptors GPR3, GPR6, GPR12, GPR18, and GPR55 [119]. Regarding NAFLD, Silvestri et al. have evidenced a dose- and time-dependent anti-hepato-steatosis effect of CBD, with a reduction in lipid accumulation both in an in vitro model using a human hepatocyte cell line, as well as in obese mice [32]. Interestingly, these hepatoprotective effects were independent of CB1R or TRPV1 activation [32]. In addition, in HFD-fed mice, CBD decreased liver inflammation via reducing the NF-κB activation and inhibiting the pyroptosis via the suppression of the activation of the NLRP3 inflammasome pathway in Kupffer cells [33]. CBD was also able to decrease liver inflammation, hepatocyte apoptosis, and oxidative stress through the inhibition of p38/JNK MAPK activation and Kupffer-cell activation, and the decreased secretion of the proinflammatory cytokine TNF-α, independently of CB1R or CB2R activation [29]. Additionally, in alcohol-fed rats and mice, CBD treatment protected the animals from alcohol-induced liver fibrosis through the selective death of activated HSC via a CB1R/CB2R independent pathway [30]. In addition, CBD treatment was also able to protect mice against alcohol-induced steatosis through similar mechanisms as the experimental NAFLD and NASH models, including the suppression of oxidative stress, the inhibition of p38/JNK MAPK activation and hepatic neutrophils infiltration, and by increasing autophagy [31,34]. Finally, in an in vitro HCV-infection model, CBD significantly inhibited the HCV replication in a dose-dependent manner [120]. Taken together, these preclinical findings suggest that CBD could have therapeutic benefits in the management of CLDs, including NAFLD, NASH, ALD, or even HCV-induced liver fibrosis. Moreover, as the hepatoprotective properties of CBD, including the antisteatogenic, antioxidant, and anti-inflammatory properties, seem to not be mediated via CB1R or CB2R, the use of CBD would not induce adverse events related to the activation of nonhepatic CB1R/CB2R; conversely, were CB2R agonists or peripherally restricted CB1R antagonists to ever be approved for the treatment of these disorders, one could envisage their coadministration with CBD as a way of inducing synergistic therapeutic actions with lower doses of the former synthetic compounds.

Apart from the major phytocannabinoids THC and CBD, other phytocannabinoids have also demonstrated potential therapeutic properties against the predictors of CLDs in preclinical studies (Table 1) [32,37,39,40,41,121]. Among these phytocannabinoids, Δ^9^- THCV is a natural nonpsychoactive analog of THC [121]. THCV acts as both a CB1R/CB2R agonist, at high doses, and, more interestingly, as a CB1R/CB2R-neutral antagonist at lower concentrations, and it can also activate GPR55 as well as TRP channels [121]. Similar to CBD, THCV has also demonstrated promising capacities in preventing the predictors of CLDs both in vitro [32] and in animal models [37,38,39]. Indeed, treating human hepatocytes with THCV reduced the intracellular lipid levels and induced lipolysis through the activation of the AMPK2a, STATs, and ERK1/2 signaling pathways [32]. In vivo, the administration of increasing doses of THCV (3, 10, and 30 mg/kg) in mice induced hypophagia and body-weight reduction [37]. In addition, THCV (2.5–12.5 mg/kg) was able to reduce the body-fat content, fasting insulin, as well as the 30 min insulin response to oral glucose tolerance in HFD-fed obese mice [39]. Moreover, the administration of a high dose (12.5 mg/kg) of THCV in genetically obese mice resulted in the reduction in liver triglycerides, thereby highlighting the antisteatogenic potential of THCV [39]. Furthermore, the combination of THCV with CBD at a 1:1 ratio (THCV/CBD; 3:3 mg/kg) was able to reduce the total cholesterol levels, liver triglycerides, and fasting insulin, and increased the HDL-C and energetic expenditure [39]. Based on these preclinical findings, THCV would constitute a promising therapeutic candidate, alone or in combination with CBD, for the management of CLDs. Another phytocannabinoid, Δ^9^-THCA-A, was also shown to reduce lipid accumulation and prevent metabolic disease in a mice model of obesity [39,40,41]. ∆^9^-THCA is proposed as a partial agonist of CB1R and CB2R, with greater affinity for CB2R, as well as an agonist of PPAR-γ [40]. In a model of HFD-fed mice, Palomares et al. showed that treating HFD-fed obese mice with Δ^9^-THCA-A was able to reduce the body-weight gain and fat accumulation, and strongly decreased the glucose intolerance and insulin resistance. Parallelly, Δ^9^-THCA-A also markedly prevented liver steatosis, adipogenesis, and macrophage infiltration in fat tissues, thereby alleviating adipose-tissue inflammation [40]. Likewise, Carmona-Hidalgo et al. also showed that Δ^9^-THCA significantly attenuated liver fibrosis and inflammation and reduced the T-cell and macrophage infiltration in the liver [41]. Interestingly, the hepatoprotective effect of THCA seems to be mediated via PPAR-γ [40]. Finally, the synthetic atypical cannabinoid Abn-CBD, a CBD derivative that does not target the canonical cannabinoid receptors CB1R and CB2R, was recently shown to exert hepatoprotective properties by lowering hepatic fibrosis, liver inflammation, and inflammatory-cell infiltration in the liver, as well as by reducing systemic inflammation and oxidative stress [42]. Based on these preclinical findings, cannabinoids, alone or in combination, have promising potential to prevent the predictors of CLDs, including liver fibrosis, steatosis, lipid accumulation, and liver inflammation, through the modulation of the peripheral or hepatic eCBS, thus providing a rationale for additional clinical human studies on their use for the management of CLDs.

Accordingly, some human clinical-trial studies have been launched to evaluate the effects of CBD and THCV, alone or in combination, on the prevention of CLDs [35,36]. Despite the promising hepatoprotective effects of CBD observed in experimental models of CLDs, all available clinical studies on humans evaluating the therapeutic effect of CBD on liver-metabolism disorders, such as diabetes, did not confirm any therapeutic benefits of CBD (Table 2) [35,36]. A phase II randomized controlled trial comparing daily CBD (200/400/800 mg) intake for 8 weeks with a placebo in people with steatosis (*n* = 25) did not show a significant reduction in the hepatic fat accumulation, total serum cholesterol, or total serum triglycerides compared with the placebo group, even at a high dose (800 mg) [36,122]. In another pilot clinical trial, where CBD (100 mg) was administrated twice daily, alone or in combination with THCV (5 mg) (total doses: CBD: 200 mg; THCV: 10 mg) in diabetic patients, no significant changes in the direct hepatic triglyceride levels, total serum cholesterol, or total serum triglycerides emerged. In addition, no effect on inflammation was observed for either CBD or THCV, alone or in combination [35]. However, some indirect effects on the indicators of glucose or lipid metabolism were detected, and particularly for THCV [35]. Indeed, when administered alone, CBD induced a significant reduction in the concentration of resistin (an adipocyte-secreted hormone associated with insulin resistance) when CBD-treated participants were compared with themselves from baseline to the end of the follow-up, thereby suggesting the potential of CBD to reduce insulin resistance in these diabetic patients. However, this effect was not observed when comparing the CBD-treated group to the placebo group. With regard to THCV, it had no direct effect on the HDL-C concentration, but it increased the concentration of the apolipoprotein A, one of the main components of HDL-C. Similarly, THCV treatment also produced indirect effects associated with the regulation of glucose metabolism. Indeed, THCV treatment reduced the fasting plasmatic glucose level, significantly improved the 3 h blood glucose response, and increased the function of pancreatic HOMA2 β-cells, suggesting an indirect effect on insulin secretion [35]. Furthermore, THCV treatment also induced a significant increase in the adiponectin level when compared with the placebo group, indicating the indirect impact of THCV treatment on lipid and glucose metabolism [35]. Finally, the combination of CBD with THCV seemed to overshadow the positive effect observed for THCV [35]. The findings of this pilot clinical trial did not support the hepatoprotective effect of CBD observed in the various preclinical studies, although they raised interest in the promising potential of THCV in the management of liver diseases associated with a dysregulation of glucose or lipid metabolism. Additional studies are thus needed to fully elucidate the discrepancy observed between the preclinical and clinical data on the efficacy of CBD as a therapeutic candidate for alleviating NAFLD, and to better understand and potentiate the positive effects observed with THCV treatment.

The discrepancies between the preclinical and clinical studies underscore the need to consider different possible reasons for their occurrence. The success rate of drug development is only 10–15% [123,124,125]. This discrepancy may exist, in part, due to the proportion of patients with advanced liver fibrosis in clinical studies, where side effects or drug-induced liver injury may be significant issues. As reviewed by Sun et al., three of the most common reasons underlying drug clinical failure, despite favorable preclinical data, include a lack of clinical efficacy, unmanageable toxicity, and poor drug-like properties [126]. The success/failure of a drug depends on the delicate balance among the clinical dose, the efficacy in disease-targeted organs, and toxicity in healthy organs [126]. It is possible that optimization efforts may overemphasize one aspect while overlooking another [126].

The drug metabolism in the human body can also be an important reason for the differences between preclinical and clinical studies in humans that could affect the clinical efficacy of a drug [126]. Because cannabinoids are generally hepatically metabolized [127], particular attention to the safety and dosage of these drugs in people suffering from CLD should be considered. Indeed, several phytocannabinoids, including CBD or THC, are metabolized by the Cytochrome P450 (CYP450) system in the liver, and particularly by the CYP2C9 and CYP3A4 isoforms [127]. Many studies have underlined the deleterious effect of advanced liver diseases on both the expression and activity of these CYP isoforms, which are significantly decreased in cirrhotic livers, resulting in the alteration of the clearance of some drugs metabolized by these liver enzymes [128]. Thus, in people with CLDs, the reduced activity of the CYP system could affect the cannabinoid metabolism, thereby reducing their bioavailability and making them ineffective, or even driving toxicity by increasing their concentration and worsening the disease. Conversely, the impaired hepatic metabolism of cannabinoids may reduce the production of their oxidative metabolites, which may act as the mediators of some cannabinoid pharmacological actions. Moreover, as people with CLDs are generally experiencing several other pathologies requiring a polypharmacy, a risk for pharmacokinetic drug–drug interactions could exist, which may explain, in part, the ineffectiveness of CBD treatment in clinical trials. Indeed, many drugs have been shown to either upregulate or downregulate the activity of the CYP450 system, which may then affect the hepatic metabolism of the cannabinoids by reducing or increasing their bioavailability. For example, ketoconazole, an antifungal with inhibitory properties on the CYP3A4, has been shown to increase the peak concentrations of both THC and CBD by about twofold, while other drugs, such as the tuberculosis medication rifampin, a CYP3A4 inducer, significantly reduced the plasma levels of THC and CBD [129]. It has also been shown that CBD can inhibit the activity of the CYP2C9, and therefore, it could also affect the metabolism of other drugs, making them ineffective or even toxic [130]. These findings raise the need to further evaluate the safety and dosing strategy for cannabinoids in an optimal clinical-trial design in the specific context of people with CLD and polypharmacy for other pathologies. Besides NAFLD, to the best of our knowledge, there is no clinical trial available that assesses the impact of CBD or other cannabinoids on alcohol-induced liver disease or liver complication induced by chronic HCV infection.

## 5. Conclusions and Future Perspectives

Given the significant contribution of the hepatic eCBS and its downstream pathways in the regulation of liver metabolism and the setting of liver abnormalities, pharmacologically targeting peripheral CBRs may have promising potential therapeutic benefits for the treatment of CLDs [72]. Besides this, the use of cannabis by people at risk of developing chronic liver disorders has also suggested hepatoprotective effects by reducing the frequency of NAFLD, ALD, or HCV-induced liver disorders, which would suggest that cannabinoid-based medicine may be effective in treating CLDs [72]. However, human clinical trials using purified CBD have unfortunately shown no beneficial effect on lipid metabolism disorders in diabetic people at risk for NAFLD [35,36]. Thus, the beneficial effects on liver disorders observed in people taking cannabis might be attributable to the other less studied phytocannabinoids, such as THCV [35,38], CBDA, or THCA [40,41], as highlighted by the preclinical and THCV-based clinical trials [35], or even to noncannabinoid metabolites, such as β-caryophyllene. Moreover, these hepatoprotective effects could be mediated through cannabinoid receptors other than the classical CB1R and CB2R, as demonstrated in experimental models of CLDs [72]. Therefore, expanding the research on these less studied phytocannabinoids and their synthetic derivatives, such as Abn-CBD [42], with a focus on their mode of action on liver metabolism, might provide promising advances in the development of new and original therapeutics for the management of liver disorders, such as NAFLD, ALD, and HCV-induced liver complications.

## Figures and Tables

**Table 1:** Preclinical and clinical studies assessing the effect of cannabinoid-based medicine on chronic liver diseases.

Main Outcomes and Conclusions	Study Design and Methodology	References
**Cannabidiol**		
CBD significantly reduced liver inflammation, oxidative/nitrative stress, and cell death, and also attenuated the bacterial endotoxin-triggered NF-κB activation and TNF-α production in isolated Kupffer cells; CBD reduced the expression of adhesion molecules in primary human liver sinusoidal endothelial cells stimulated with TNF-α, and the attachment of human neutrophils to the activated endothelium; Protective effects were preserved in CB2 knockout mice and were not prevented by CB1/2 antagonists in vitro.	In vivo mice model of segmental hepatic ischemia.	[29]
CBD selectively elicited an endoplasmic-reticulum (ER) stress response in activated hepatic stellate cells (HSCs), but not in quiescent HSCs or primary hepatocytes; CBD induced the activation of the proapoptotic IRE1/ASK1/c-Jun N-terminal kinase pathway, leading to the death of activated HSC.	In vitro and in vivo models of hepatic fibrosis.	[30]
CBD prevented acute alcohol-induced liver steatosis in mice, possibly by preventing the increase in oxidative stress and the activation of the JNK MAPK pathway; CBD increased autophagy both in vitro and in vivo; CBD prevented the decrease in alcohol-induced autophagy.	In vitro and in vivo models of alcohol-induced liver steatosis.	[31]
THCV and CBD directly reduced accumulated lipid levels in vitro in a hepatosteatosis model and in adipocytes; THCV and CBD also induced posttranslational changes in a variety of proteins associated with lipid metabolism and mitochondrial activity, such as CREB, PRAS40, AMPKa2, and several STATs, both in vitro and in vivo.	In vitro and in vivo models of hepatosteatosis. Transcriptional, posttranslational, and metabolomic assays.	[32]
CBD significantly inhibited NF-κB p65 nuclear translocation and the activation of NLRP3 inflammasome in macrophages in vivo and in vitro, leading to the reduction in liver inflammation induced by the HFC diet.	In vivo model of NAFLD with high fat diet (HFD)-fed mice for 8 weeks; In vitro model of inflammation with macrophage cell line (RAW264.7) incubated with LPS+ATP, and with or without CBD.	[33]
CBD treatment inhibited macrophage recruitment and suppressed activation of NFκB–NLRP3–pyroptosis pathway in mice livers; The hepatoprotective property of CBD might be a result of the inhibition of inflammation via the alleviation of the activation of the hepatic NFκB–NLRP3 inflammasome–pyroptosis pathway.	Mice-liver-injury model induced by ethanol plus high-fat high-cholesterol diet (EHFD) for 8 weeks.	[34]
Compared with placebo, THCV significantly decreased fasting plasma glucose and improved pancreatic β-cell function, adiponectin, and apolipoprotein A, although plasma HDL was unaffected; Compared with baseline (but not placebo), CBD decreased resistin and increased glucose-dependent insulinotropic peptide; None of the combination treatments had a significant impact on end points. CBD and THCV were well tolerated; No significant changes in the visceral adiposity or liver triglycerides assessed by MRI/MRS after treatment.	Randomized double-blind placebo-controlled study: 5 treatment arms: CBD alone (100 mg twice daily); THCV alone (5 mg twice daily); 1:1 ratio of CBD and THCV (5 mg/5 mg, twice daily); 20:1 ratio of CBD and THCV (100 mg/5 mg, twice daily); or matched placebo for 13 weeks, on 62 subjects with noninsulin-treated type 2 diabetes.	[35]
Mean liver triglyceride levels did not significantly differ between the CBD and placebo groups.	Randomized partially blind placebo-controlled dose-ranging phase 2 clinical study: 200/400/800 mg of CBD daily or placebo for 8 weeks on 25 participants with NAFLD.	[36]
**Tetrahydrocannabivarin**		
THCV induced hypophagia and weight reduction at low doses (3 mg/kg).	In vivo model of fasting and feeding mice.	[37]
Δ^8^-THCV activated human CB2R of transfected CHO cells in vitro; Δ^8^-THCV alleviated hepatic injury, and decreased proinflammatory chemokines CCL3, CXCL2, TNF-a, and the ICAM-1 level and neutrophil infiltration in vivo; CB2R antagonist attenuated the protective effects of Δ^8^-THCV, while a CBR antagonist tended to enhance it.	In vitro and in vivo mice models of hepatic ischemia.	[38]
THCV did not significantly affect food intake or body-weight gain in any of the studies, but produced an early and transient increase in energy expenditure; THCV dose-dependently reduced glucose intolerance in genetically obese mice and improved glucose tolerance and increased insulin sensitivity in DIO mice, without consistently affecting plasma lipids; THCV also restored insulin signaling in insulin-resistant hepatocytes.	In vivo mice model of diet-induced obesity (DIO) treated with regimens of increasing doses of THCV: Regimen 1: 0.3, 1, 2.5, 5, and 12.5 mg/kg of THCV orally, twice daily for 30 days; Regimen 2: 0.1, 0.5, 2.5, and 12.5 mg/kg of THCV orally, once daily for 45 days; Regimen 3: 0.3, and 3 mg/kg of THCV orally, once daily for 30 days; Regimen 4: 0.1, 0.5, 2.5, and 12.5 mg/kg of THCV orally, once daily for 30 days.	[39]
THCV and CBD directly reduced accumulated lipid levels in vitro in a hepatosteatosis model and in adipocytes; THCV and CBD also induced posttranslational changes in a variety of proteins associated with lipid metabolism and mitochondrial activity, such as CREB, PRAS40, AMPKa2, and several STATs, both in vitro and in vivo.	In vitro and in vivo models of hepatosteatosis. Transcriptional, posttranslational, and metabolomic assays.	[32]
Compared with placebo, THCV significantly decreased fasting plasma glucose and improved pancreatic β-cell function, adiponectin, and apolipoprotein A, although plasma HDL was unaffected; Compared with baseline (but not placebo), CBD decreased resistin and increased glucose-dependent insulinotropic peptide; None of the combination treatments had a significant impact on end points. CBD and THCV were well tolerated; No significant changes in visceral adiposity or liver triglycerides assessed by MRI/MRS after treatment.	Randomized double-blind placebo-controlled study: 5 treatment arms: CBD alone (100 mg twice daily); THCV alone (5 mg twice daily); 1:1 ratio of CBD and THCV (5 mg/5 mg, twice daily); 20:1 ratio of CBD and THCV (100 mg/5 mg, twice daily); matched placebo for 13 weeks, on 62 subjects with noninsulin-treated type 2 diabetes.	[35]
**Tetrahydrocannabinolic acid**		
Δ^9^-THCA-A binds to and activates PPARγ by acting at both the canonical and alternative sites of the ligand-binding domain; In HFD-induced obese mice, Δ^9^-THCA-A significantly reduced fat mass and body-weight gain, markedly ameliorating glucose intolerance and insulin resistance, and largely preventing liver steatosis, adipogenesis, and macrophage infiltration in fat tissues; Δ^9^-THCA-A caused browning of white adipose tissue (iWAT) and displayed potent anti-inflammatory actions in HFD mice.	In vitro functional assay and in vivo mice model of high fat diet (HFD)-induced obesity.	[40]
Δ^9^-THCA inhibited the expression of Tenascin C (TNC) and Col3A1 induced by TGFβ in LX-2 cells and the transcriptional activity of the Col1A2 promoter in fibroblasts; Δ^9^-THCA significantly attenuated CCl4-induced liver fibrosis and inflammation and reduced T-cell and macrophage infiltration; Δ^9^-THCA significantly reduced body weight and adiposity, improved glucose tolerance, and drastically attenuated liver fibrosis due to diet-induced obesity and immune-cell infiltration.	In vitro model of liver fibrosis and in vivo ice model of nonalcoholic liver fibrosis induced by CCl4 treatment of 23-weeks of high-fat-diet (HFD) feeding.	[41]
**Atypical Cannabinoid Abn-CBD**		
Abn-CBD reduced hyperinsulinemia and markers of systemic low-grade inflammation in plasma and fat, also promoting WAT browning; Abn-CBD lowered pancreatic islets apoptosis, inflammation, and oxidative stress, and promoted beta-cell proliferation; Abn-CBD lowered hepatic fibrosis, inflammation, and macrophage infiltration.	Diet-induced obese mouse model of prediabetes and nonalcoholic fatty liver disease (NAFLD).	[42]

**Table 2:** Studies assessing the effect of cannabis smoking on NAFLD/NASH, ALD, and HCV/HBV-induced liver disorders.

Main Outcome	Study Design	Participant Characteristics	Method to Assess Outcome	Reference
**NAFLD/NASH**				
Cannabis use is associated with lower prevalence of NAFLD/NASH.	Population-based case–control study	A total of 5,950,391 patients, 18 years and older, in three groups: noncannabis users (98.04%), nondependent cannabis users (1.74%), and dependent cannabis users (0.22%).	Multivariate logistic regression to determine the odds of developing NAFLD with respect to cannabis use.	[83]
Cannabis use is associated with lower levels of fasting insulin and insulin resistance.	Examination survey	4657 adults aged 18 years and older	Fasting insulin and glucose measured via blood samples after a 9 h fast, and HOMA-IR calculated to evaluate insulin resistance. Multiple linear regression to determine associations.	[84]
Prevalence of obesity is lower in cannabis users than in nonusers.	Cross-sectional data on 2 population-based nationally representative studies.	52,375 adults aged 18 years or older	Logistic regression model with obesity as a categorical outcome, and the frequency of cannabis use in the past year as the primary association.	[85]
Inverse association between cannabis use and obesity.	Population-based 3-year prospective study.	43,093 adults aged 18 years or older	General linear modeling yields estimates for change in body-mass index regressed on cannabis-use status.	[86]
Current marijuana use is associated with lower odds of metabolic syndrome across emerging and middle-aged adults.	Population-based 5-year prospective study	8478 adults, 20–59 years old	Metabolic syndrome was defined as ≥ 3 of the following: elevated fasting glucose, high triglycerides, low high-density-lipoprotein cholesterol, elevated systolic/diastolic blood pressure, and increased waist circumference. An age-stratified analysis was conducted to examine the relationship between marijuana use and metabolic syndrome among emerging adults (20–30 years), adults (31–44 years), and middle-aged adults (45–59 years).	[87]
Marijuana use was independently associated with a lower prevalence of diabetes mellitus.	Cross-sectional study	10,896 adults, 20–59 years old	Univariate and multivariate logistic regression analyses were used to determine the relationship between diabetes mellitus and marijuana use.	[88]
Active marijuana use provided a protective effect against NAFLD independent of known metabolic risk factors.	Cross-sectional data from 2 National Health and Nutrition Examination Surveys	22,366	NAFLD was defined either by a serum alanine aminotransferase (ALT) that was >30 IU/L for men and >19 IU/L for women in the absence of other liver diseases, or based on ultrasonography.	[89]
**ALD**				
Among alcohol users, cannabis use was associated with significantly lower odds of developing alcoholic steatosis, steatohepatitis, fibrosis, cirrhosis, and hepatocellular carcinoma.	Cross-sectional data from National Health and Nutrition Examination Survey	319,514 adults 18 years and older	Univariate and multivariate logistic regression analyses were used to determine the relationship between alcoholic steatosis, steatohepatitis, fibrosis, cirrhosis, hepatocellular carcinoma, and cannabis use.	[90]
No association between cannabis use and advanced liver fibrosis in heavy alcohol drinkers.	Cross-sectional study	248 HIV-positive individuals with heavy alcohol use	Transient elastography was used to detect advanced liver fibrosis among participants.	[91]
**HCV/HBV infection**				
Regular or daily cannabis use was associated with a reduced risk of an elevated fatty liver index in HIV–HCV-coinfected individuals.	5-year longitudinal study	997 HIV–HCV-coinfected individuals	Mixed-effects multivariable logistic and linear regression models	[92]
Cannabis use was associated with lower risks of obesity in chronically HBV-infected individuals.	Cross-sectional study	3706 chronically HBV-infected individuals	Logistic and multinomial regression analyses	[93]
Cannabis use was independently associated with a lower risk of diabetes in chronically HCV-infected individuals.	Cross-sectional study	10,445 chronically HCV-infected individuals	Multivariate logistic regression analyses	[94]
Daily cannabis use was independently associated with a reduced prevalence of steatosis.	Cross-sectional study in a nationwide multicenter cohort	838 adults, HIV–HCV-coinfected individuals	A logistic regression model was used to evaluate the association between cannabis use and steatosis.	[95]
THC-rich cannabis use was not associated with progression to significant liver fibrosis.	11-year longitudinal study	575 HIV–HCV-coinfected women	Cox proportional hazards regression analysis	[96]
Cannabis use is associated with a lower insulin-resistance risk in HIV–HCV-coinfected individuals.	60-month longitudinal study	703 HIV–HCV-coinfected individuals	A mixed-effects multivariable logistic regression model	[97]
Daily cannabis smoking is significantly associated with fibrosis progression during chronic hepatitis C virus infection.	Cross-sectional study	270 untreated chronically hepatitis-infected individuals	Multivariate stepwise logistic regression analyses	[98]
Daily cannabis smoking as a novel independent predictor of steatosis severity during chronic hepatitis C virus infection.	Cross-sectional study	315 untreated chronically hepatitis-infected individuals	Multivariate stepwise logistic regression analyses	[99]
